# Study on Dynamic Column Behavior and Complexation Mechanism of DBS-Modified Crown Ether-Based Silica to ^90^Sr

**DOI:** 10.3390/toxics11110919

**Published:** 2023-11-10

**Authors:** Yan Wu, Hongji Sang, Jiawei Zheng, Lejin Xu, Tong Liu, Yuezhou Wei

**Affiliations:** 1 School of Nuclear Science and Engineering, Shanghai Jiao Tong University, Shanghai 200240, China; 2 School of Energy and Power Engineering, Huazhong University of Science and Technology, Wuhan 430074, China

**Keywords:** strontium, crown ether, DBS, column separation, dynamic simulation

## Abstract

A crown ether-loaded hybrid adsorbent suitable for the separation and enrichment of strontium from high-level liquid waste was synthesized. 4′,4′(5″)-di(tert-butylcyclohexano)-18-crown-6 (DtBuCH18C6) and its modifiers dodecyl benzenesulfonic acid (DBS) and 1-dodecanol were impregnated into silica-based polymer support. The hybrid adsorbent exhibited excellent Sr(II) selectivity ability, and effective chromatographic separation and recovery of Sr(II) from simulated high-level liquid waste could be achieved with a (DtBuCH18C6 + DBS + dodec)/SiO_2_-P packed column. The recovery rate of Sr(II) calculated based on the mass balance was approximately 99% and over 80% for the other coexisting metal ions. An appropriate increase in the concentration of Na-DTPA eluent was favorable to improve the efficiency of the elution process because of the increased complexation capacity of [DTPA]^5−^ to Sr(II). The developed theoretical model can simulate the dynamic breakthrough curves of the material on the basis of short column data, thereby predicting the scale-up column of the practical operation. Density functional theory calculation was used to explore the action mechanism of DBS modifiers on the Sr(II) complexation process of crown ether groups. Two Sr(II) complexation isomeric models of DtBuCH18C6 were established, and the calculation results revealed a similar complexation ability. DtBuCH18C6 could form a stable Sr(II) complexation structure with DBS coordination, which further indicated that DBS could be a ligand to promote the Sr(II) adsorption ability of crown ether materials.

## 1. Introduction

Over the last decades, the rapid development of the nuclear energy industry has increased the demand for the enhanced efficiency of high-level liquid waste (HLLW) treatment [1]. As a common β-emitter nuclide within HLLW, ^90^Sr has a long half-life and a high heat release rate, thereby possibly weakening the mechanical stability of glass curing products and complicating the long-term geological disposal process of HLLW [2,3,4]. ^90^Sr removal from HLLW offers the benefits of decreasing the cost of geological disposal, improving the utilization of the repository space, and reducing the threat of radiation to the ecosystem and humans [5]. Therefore, ^90^Sr decontamination of HLLW is of great significance.

Various techniques have been reported for the application of Sr(II) removal from radioactive wastewater, including adsorption [6,7,8], ion exchange [9,10,11], extraction techniques [12,13], and membrane separation [14]. The extraction method possesses the advantages of high selectivity and recovery and is suitable for continuous operation under high-level radiation conditions. Therefore, it has been proposed as the primary method for large-scale Sr(II) decontamination. However, the extraction process, especially the steps of solvent extraction, consistently suffers the drawbacks of being complicated, requiring multiple steps, having a high organic phase consumption, and resulting in secondary waste generation as a result of solvent hydrolytic and radiolytic degradation [15,16]. Thus, the novel technology of extraction chromatography, which is based on solid adsorbent and has the advantages of simple handling and good mechanical and radiation resistance of the material, is gaining more attention [17].

Several solid adsorbents that integrate the organic extractant with solid supports to the application of Sr(II) extraction chromatography separation have been reported [18,19,20]. Considering the strong selectivity chelation of crown ether compounds with alkaline earth metal cations, solid-extraction adsorbent with crown ether as the adsorption phase has become an attractive option. 4′,4′(5″)-di(tert-butylcyclohexano)-18-crown-6 (DtBuCH18C6), which polyether cavity size matches the Sr(II) ionic radius, possesses superior hydrolytic and radiation stability and can rapidly chelate with Sr(II) inside the aqueous solution, yielding a complex with strong stability. Therefore, it has become an efficient adsorption phase for the removal of ^90^Sr from HLLW [21]. In our previous work, a novel crown ether solid-extraction adsorbent was successfully synthesized by impregnating DtBuCH18C6 into the macroporous silica-based polymer composite carrier (SiO_2_-P) [22]. The synthesized adsorbent exhibited excellent mechanical properties and chemical and radiation resistance, which was suitable for column separation. Moreover, 1-dodecanol and dodecyl benzenesulfonic acid (DBS) were added as the molecule modifiers to further widen the adsorbent applicable acidity range, and the adsorbent containing DBS better promoted the adsorption of Sr(II) compared to the DBS-free one, particularly addressing the defect of poor adsorption capacity in low-acidity conditions [23]. The fundamental adsorption performance of adsorbent with DBS modification has been investigated in the literature, demonstrating that DBS works as a counter ion. However, DBS modification significantly improves the Sr(II) adsorption ability of the adsorbent, leading to difficulties in desorption using deionized water. The dynamic Sr(II) adsorption behavior of the adsorbent applied in the column separation process remains poorly understood. Thus, an adsorbent synthesized with DBS modification needs to be further investigated.

Other aspects we would like to explore are how DtBuCH18C6 supplies the Sr(II) selective separation ability within the nitrate system and how the DBS molecule modifiers influence the complexation process. In general, the metal ion separation ability of crown ethers could be explained by the compatibility of the polyether cavity size with the cation size and the hard and soft acids and bases principle—namely, the stability of the yielded complex mainly depends on the nature of the electron donors and the cation speciation in the aqueous solution [24,25]. Several experimental investigations have demonstrated that the principle of Sr(II) adsorption is mainly controlled by the interaction of Sr(II) with the oxygen in crown ethers to form Sr-O bonds [23,26]. Some theoretical studies have revealed the complexation mechanism of crown ethers with Sr(II) during the extraction process [27,28,29]. However, nitrate has been identified as the possible ligand that participates in the reaction of Sr(II) extraction by DtBuCH18C6 from the solutions; the same phenomenon may be observed in DBS [30,31]. Thus, the extraction mechanism of DtBuCH18C6 to Sr(II) with DBS as the ligand needs to be further theoretically studied. In addition, the scaling up of column experimental studies is necessary before adsorbents can be used industrially. However, multiple expensive and time-consuming pilot-scale experiments are usually required to obtain dynamic experimental results as accurately as possible. Therefore, analytical models such as Thomas, the Clark column, and the Wolborska model have been used to evaluate the dynamic adsorption performance of materials. These models are mainly used for fitting experimental data and can hardly accurately infer the effects caused by changes in operating conditions. Therefore, the establishment of a numerical calculation model for breakthrough curve simulations is crucial for optimizing the design of dynamic adsorption processes.

In this study, a DBS-modified crown adsorbent was prepared. We constructed the Sr(II) adsorption modeling framework of DtBuCH18C6 on the basis of the optimized electrostatic potential (ESP) and calculated the complexation of DtBuCH18C6 to Sr(II) with DBS coordination to explore the possible promotion mechanism of DBS decoration to Sr(II) adsorption of a hybrid crown adsorbent, with a view toward providing a theoretical basis for the design of more efficient adsorbent materials. Further, we investigated the dynamic breakthrough property of the adsorbent, and a theoretical model was established for the breakthrough curve prediction. A mechanistic model was chosen to theoretically describe the adsorption process with a mass conservation equation, kinetic equation, and adsorption isotherm. Approaches for scale-up techniques applied to the removal of ^90^Sr from HLLW were established. Four reagents were utilized for the investigation of the adsorbent Sr(II) elution capacity, and dynamic column separation of Sr(II) from the simulated HLLW was conducted with the best eluent for further performance evaluation.

## 2. Experiment and Calculation

### 2.1. Preparation of Material

**Figure 1:**
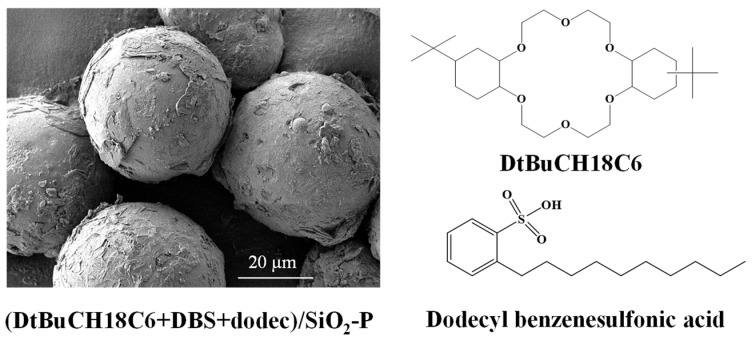
SEM and chemical structure of (DtBuCH18C6 + DBS + dodec)/SiO_2_-P.

The (DtBuCH18C6 + DBS + dodec)/SiO_2_-P adsorbent (Figure 1) with DBS modification was synthesized following the rotary vacuum evaporation method [23]. DtBuCH18C6 (25.5 wt%) and DBS were dissolved in dichloromethane and then mixed with SiO_2_-P particles in a glass flask and then stirred gently by a rotary evaporator. The SiO_2_-P particles were synthesized by immobilizing a 20% styrene–divinylbenzene (SDB) copolymer into the SiO_2_ porous. Sr(NO_3_)_2_ was of reagent grade and purchased from Sinopharm Chemical Reagent Co., Ltd, Shanghai, China. Diethylenetriaminepentaacetic acid (DPTA) and diethylenetriaminepentaacetic acid pentasodium salt solution (Na-DTPA) were purchased from Sinopharm Chemical Reagent Co., Ltd, Shanghai, China. The simulated HLLW (SHLLW) containing 3 M HNO_3_ was prepared, and the main initial concentration of SHLLW is summarized in Table 1.

### 2.2. Batch Experiments

Static batch experiments, which included the Sr(II) adsorption selectivity estimate and desorption property evaluation, were performed using a thermostatic water bath shaker (Tokyo PIKAKIKA Co., Ltd., Tokyo, Japan) at 25 °C for 24 h. In our previous study, the adsorption of Sr(II) by (DtBuCH18C6 + DBS + dodec)/SiO_2_-P attained equilibrium within 5 h [23]; thus, the 24 h shaking time was sufficient. A total of 50 mg (DtBuCH18C6 + DBS + dodec)/SiO_2_-P was added to an aqueous solution (5 mL). The equilibrium distribution coefficient (*K_d_*, mL/g) and the elution rate (*E_r_*, %) were calculated using the following equations:

(1) $$K_{d}=\frac{C_{0}-C_{e}}{C_{e}}\times \frac{V}{m}$$

(2) $$E_{r}=\frac{C_{d}}{Q_{e}}\times \frac{V}{m}\times 100\%$$

where *C*_0_ and *C_e_* (mg/L) denote the concentrations of metal ions inside the aqueous solutions before and after equilibrium adsorption, respectively. *V* (mL) and *m* (g) represent the volume of aqueous solution and mass of the adsorbent, respectively. *C_d_* (mg/L) denotes the metal equilibrium concentration of Sr(II) inside the elution solution, and *Q_e_* (mg/g) is the adsorption capacity of the adsorbent.

### 2.3. Dynamic Column Operation

A series of dynamic column adsorption experiments was conducted using a glass column of *ϕ*10 mm × *h* 100 mm that was packed with around 2 g of (DtBuCH18C6 + DBS + dodec)/SiO_2_-P adsorbent and then fed with different solutions. The breakthrough curve was determined by supplying the Sr(II) aqueous solution (5 mM Sr in 3 M HNO_3_) at a flow of 0.3 mL/min. Eluent with three concentrations of 0.05, 0.075, and 0.1 mol/L was fed at a feeding rate of 0.5 mL/min to further investigate the dynamic Sr(II) desorption property of the adsorbent. The chromatographic column separation of Sr(II) was prepared with 3 mL SHLLW feeding, followed by 50 mL of 3 M HNO_3_ and 120 mL of 0.1 M Na-DTPA solutions. A fraction collector was used for effluent collection, and concentrations of various metal cations were measured by using a sequential plasma spectrometer (ICP-AES, Shimadzu Corporation, Kyoto, Japan).

### 2.4. Mechanistic Model of Column Adsorption

A theoretical model based on the mass balance principle, adsorption Freundlich model, and adsorption kinetic model was developed by our group to describe dynamic column separation processes [32]. The model assumed that (a) the solution flowed through with a constant velocity, (b) the density of the adsorbate did not change during the adsorption process, and (c) the temperature inside the column remained uniform and identical.

Thus, the adsorption process inside the column bed can be represented using Equations (3) and (4):

(3) $$\frac{\partial C}{\partial t}=\frac{E}{\varepsilon }\frac{\partial^{2}C}{\partial z^{2}}-\frac{u}{\varepsilon }\frac{\partial C}{\partial z}+\frac{r_{a}}{\varepsilon }$$

(4) $$\frac{\partial q}{\partial t}=-\frac{r_{a}}{m}$$

The external mass coefficient *K_fa_* was employed to summarize the factors that control the adsorption rate, and the different components inside the adsorption resin with different adsorption rates were considered, as exhibited in Equation (5).

(5) $$r_{a}=-\overset{\underset{l}{k=1}}{\sum }K_{fak}\left(C-C_{ek}\right)$$

where values of *k* and *l* are positive.

Based on Equation (5), the kinetic model can be obtained as follows:

(6) $$\frac{\partial q_{k}}{\partial t}=\frac{K_{fak}}{m}\left(C-C_{ek}\right)$$

The Freundlich isotherm, which can accurately describe the adsorption mechanism, was chosen to be the adsorption equilibrium model, which is given as follows:

(7) $$q_{ek}=K_{Fk}C_{ek}^{b_{k}}$$

The difference method was utilized on the right side of Equation (1) for the numerical calculations. The *L* (m) column bed along the flow direction was divided into *n* stages, with each stage having a height of Δ*z* = *L*/*n*. The feeding liquid in each stage was simulated by the layer sequence model with the n-stage fully mixed.

(8) uΔz/E≅2

The efficiency of the calculation was improved by considering the value of Δ*z* to be similar to the adsorbent particle diameter *d_P_*.

The final model is described as follows:

(9) $$\frac{dC_{i}}{dt}=-\frac{u}{\varepsilon }\frac{\left(C_{i}-C_{i-1}\right)}{\Delta z}-\overset{\underset{l}{k=1}}{\sum }\frac{K_{fak}}{\varepsilon }\left(C-C_{ek}\right)$$

(10) $$\frac{\partial q_{ik}}{\partial t}=\frac{K_{fak}}{m}(C_{i}-C_{iek})$$

(11) $$C_{iek}=(q_{ik}/K_{Fk}{)}^{1/b_{k}}$$

and the initial and boundary conditions are defined as follows:

(12) $$t=0:C_{i}=0,q_{i}=0(i=1,2,\cdots ,n)$$

(13) $$i=1:C_{i-1}=C_{0}$$

where *C* (mg/L) is the concentration of Sr(II) in the solutions, *t* (h) is the time, *u* (m/h) is the flow rate, *ε* is the bed porosity, *z* (m) is the axial height along the column length, *m* (kg/m^3^) is the density of the adsorbent, *k* and *l* are positive constants, *K_fa_* is the external mass coefficient, and *K_F_* is the constant of the Freundlich model.

### 2.5. Density Functional Theory Calculation

The optimized structures and interaction energies of DtBuCH18C6 coordinated with Sr(II) in the presence and absence of the DBS modifier or nitrate ions were calculated using the m06-2x density functional as implemented in the Gaussian 16 program package [33,34]. During the process of complex geometry optimization and the frequency calculation, the SDD basic set was used for Sr(II), and the rest of the atoms were performed at the def2-svp basic set level. The component of “quantitative analysis of molecular surface” in Multiwfn 3.8 was used to calculate the electrostatic potential of the optimized models in conjunction with VMD 1.9.3 to depict the electrostatic potential (ESP) pictures. Single-point energy calculation was performed with def2-tzvp in conjunction with a popular recessive solvent (COSMO, water) model for all the elements, and D3 dispersion correction was added to describe weak interactions. The correction of the zero-point energy and the basis set superposition error correction (BSSE) were involved in the calculation of the interaction energies, and the expression is as follows [35]:

(14) $$\Delta E={E_{c}}_{omplex}-E_{DtBuCH18C6}-E_{Sr}+{E_{B}}_{SSE}$$

where Δ*E* represents the interaction free energy of the ion–crown ether complex. *E_complex_*, *E_DtBuCH_*_18*C*6_, and *E_Sr_* represent the energies of the ion–crown ether complex, DtBuCH18C6, and Sr(II) ion, respectively. *E_BSSE_* represents the BSSE energy.

## 3. Results and Discussion

### 3.1. Static Batch Experiments

#### 3.1.1. Influence of Coexisting Ions on Sr(II) Separation

**Figure 2:**
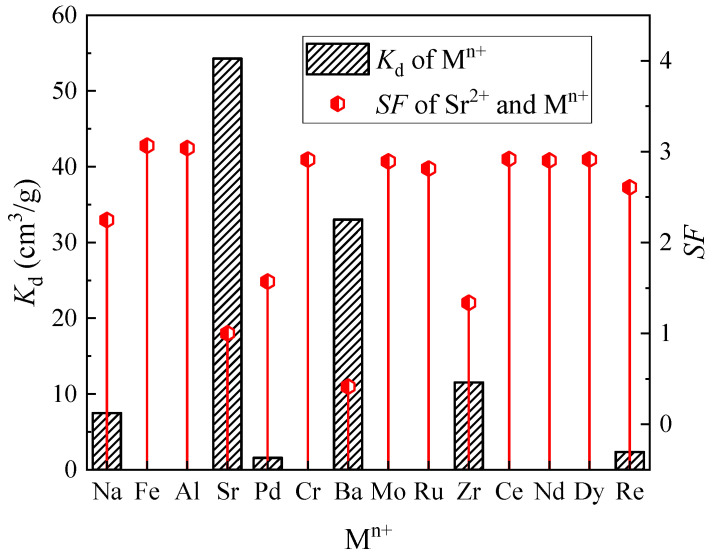
Adsorption selectivity of metal ions for (DtBuCH18C6 + DBS + dodec)/SiO_2_-P in the SHLLW.

The SHLLW with an acidity of 3 M HNO_3_ was utilized for evaluating the adsorption selectivity of the (DtBuCH18C6 + DBS + dodec)/SiO_2_-P adsorbent, and the results are summarized in Figure 2. The separation factor (SF) was determined as *K_d_,_Sr_*/*K_d_,_M_^n+^*. The *K_d_* of Sr(II) was higher than that of the other coexisting ions by up to 54.29 mL/g, thus indicating the excellent Sr(II) separation property of the adsorbent. As a result of the selective adsorption capacity of crown ether being mainly controlled by the matches of the polyether cavity size with metal cations, the removal of the adsorbent to Ba(II) and Na(I) ions could be explained with the similar size of them to Sr(II), indicating the effective competition between Ba(II) and Na(I) in the Sr(II) separation process of the adsorbent [36,37]. The calculated *K_d_* of the adsorbent to Zr(IV) was 11.53 cm^3^/g. Considering the coexistence of Mo(V) inside the HLLW, the decreased concentration of Zr(IV) could be attributed to the coprecipitation of Zr(IV) with Mo(V) to form the Zr(MoO_4_)_2_∙2H_2_O precipitate [38,39]. As a result, the adsorbent exhibited significant Sr(II) separation property from the simulated HLLW, indicating that (DtBuCH18C6 + DBS + dodec)/SiO_2_-P possessed excellent Sr(II) adsorption selectivity ability.

#### 3.1.2. Elution Behavior of Adsorbent with Different Eluents

**Figure 3:**
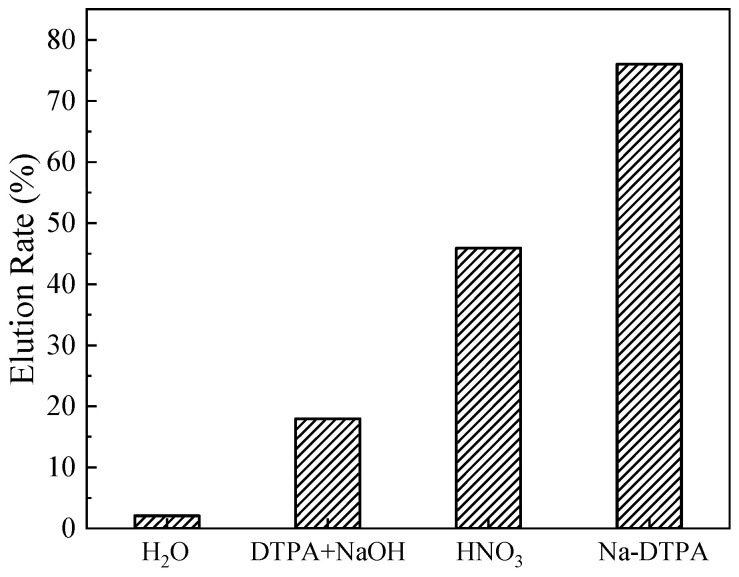
Effect of various solutions on the elution rate of Sr(II).

The Sr(II) elution rates of various eluents to (DtBuCH18C6 + DBS + dodec)/SiO_2_-P are shown in Figure 3. Compared to the deionized water, nitric acid, and DTPA+NaOH mixture, solutions of Na-DTPA exhibited the best recovery rate, up to 76.03%. According to our previous investigation, the adsorbed Sr(II) onto DtBuCH18C6-loaded resin could be eluted with deionized water [22]. However, H_2_O showed an invalid elution ability with regard to the DBS-modified adsorbent. In addition, 1 mol/L HNO_3_ recovered only 45.90% of the adsorbed Sr(II) onto the (DtBuCH18C6 + DBS + Dodec)/SiO_2_-P adsorbent, and the low Sr(II) recovery rate would result in the poor reuse performance of the material. The elution behaviors of the deionized water and nitric acid could be explained as follows: the DBS modifier could offer the coordination anion for the Sr(II) complexation of DtBuCH18C6, thus leading to the low desorption efficiency of the deionized water and nitric acid to the DBS-modified adsorbent [30].

As a commonly used metal chelate, DTPA could coordinate with Sr(II) in the solutions to form an annular structure, thus occupying the Sr(II) reaction sites and preventing the complexation reactions of Sr(II) with adsorbents [40]. The main coordination group of DTPA to metal ions is [DTPA]^5−^, which suggests that the agents of DTPA and Na-DTPA can be used for Sr(II) elution of the (DtBuCH18C6 + DBS + dodec)/SiO_2_-P adsorbent. For the mixed solution of DTPA with NaOH, NaOH was added to increase the dissolution of DTPA and adjust the main chelate ion concentrations inside the solution. However, the elution efficiency of Na-DTPA was higher than that of the DTPA+NaOH mixture. This phenomenon could be ascribed to the pH value of the DTPA+NaOH mixture solution, which was 3.6, lower than the best Sr(II) complexation condition of pH = 12. The ionic concentration of [DTPA]^5−^ inside the Na-DTPA solution was higher than DTPA + NaOH, which could offer more chelate sites for Sr(II) complexation. As a result, Na-DTPA exhibited excellent Sr(II) elution performance, which could be used as the eluent for the dynamic desorption property investigation of the (DtBuCH18C6 + DBS + dodec)/SiO_2_-P adsorbent.

### 3.2. Dynamic Column Adsorption and Simulation

#### 3.2.1. Breakthrough Curves

Given its advantages of high separation efficiency and large volumes of batch treatment capacity, the application of extraction chromatography to the region of HLLW decontamination has attracted increasing attention. However, the successful development of a chromatography method of Sr(II) separation from the HLLW needs the ion dynamic behavior inside the column bed to be described accurately. Thus, the dynamic adsorption performance of the (DtBuCH18C6 + DBS + Dodec)/SiO_2_-P adsorbent to Sr(II) at a flow rate of 0.3 mL/min was investigated. The results were simulated through the developed theoretical model.

**Figure 4:**
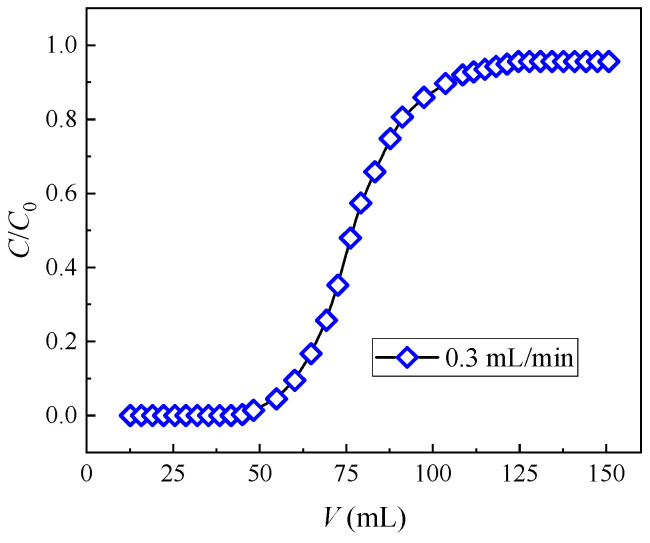
Experimental breakthrough curves of Sr(II) from the (DtBuCH18C6 + DBS + dodec)/SiO_2_-P column.

Figure 4 presents the breakthrough curve of (DtBuCH18C6 + DBS + dodec)/SiO_2_-P as the function of the feeding volume. The plots exhibit a similar trend to the typical S-shaped line, and the values of *C*/*C_o_* increase rapidly to 0.8 and then slowly reach 1. This finding demonstrates the excellent dynamic adsorption property and high efficiency of the adsorbent. The breakthrough point of 5% in column experiments is often used as a momentous indicator for evaluating the adsorption performance of adsorbents. The breakthrough capacity (B. Cap.) and total capacity (T. Cap.) were acquired when the Sr(II) concentration in the effluent reached 5% (*C*/*C_o_* = 5%) and 100% (*C*/*C_o_* = 100%) of the initial pumping concentration, respectively. Table 2 lists the column parameters, including the breakthrough volume, dynamic adsorption amount, and calculated utilization rate of the adsorption column. A relatively high column utilization rate (5% B. Cap./T. Cap.) of 88.17% was obtained, indicating the effective removal of Sr(II) by the (DtBuCH18C6 + DBS + dodec)/SiO_2_-P packed column.

#### 3.2.2. Simulation of Breakthrough Curves

**Figure 5:**
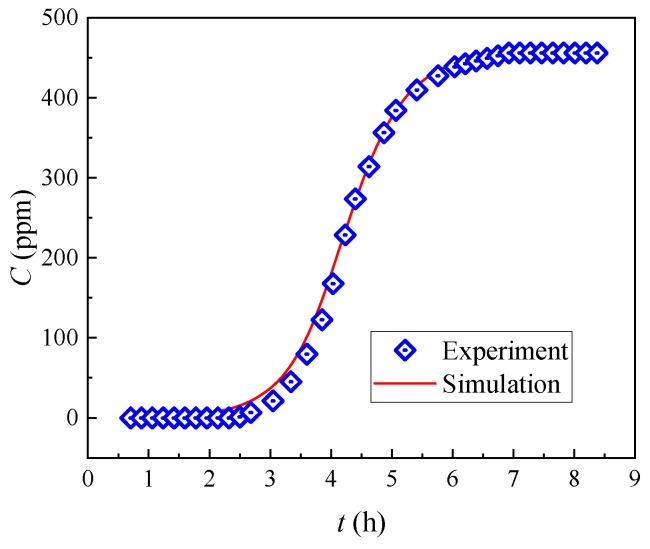
Predicted breakthrough curves of Sr(II) from the (DtBuCH18C6 + DBS + dodec)/SiO_2_-P column.

As shown in Figure 5, the 5% breakthrough period of 0.3 mL/min was 3.05 h. Based on the dynamic adsorption results of (DtBuCH18C6 + DBS + dodec)/SiO_2_-P under the considered flow rate used in designing the adsorption column theoretical model, the involved coefficient *K_fa_* of the theoretical model was calculated using Equation (15), and the adsorption isotherm parameters were obtained through Freundlich model fitting [23]. The calculated parameters of the built final column theoretical model are listed in Table 3.

(15) $$K_{fa}=\frac{u}{\Delta z}\left\{{\left[\frac{Cn(t=0)}{Co}\right]}^{-(1/n)}-1\right\}$$

where *C_n_* (*t* = 0) (g/cm^3^) represents the Sr(II) concentration of the effluent at the beginning phase of the column experiment, and Δ*z* represents the minute height of the column height division (m).

In accordance with the Runge–Kutta numerical methods, in conjunction with the column adsorption experiment conditions, the equations of the developed theoretical model were resolved. The comparison of the model fitting curves with the experimental data is shown in Figure 5. The theoretical model curves were consistent with the experimental breakthrough curves, suggesting that the numerical model could feasibly predict the dynamic adsorption process. The close values of the 5% and 100% breakthrough periods obtained through the model fitting and the experiment data further proved the appropriate model development and parameter calculations (Table 4). The similar results of prediction and experimental data at 0.5 mL/min in our previous work also proved the feasibility of a reasonable mechanical model and appropriate calculations of the parameters.

**Figure 6:**
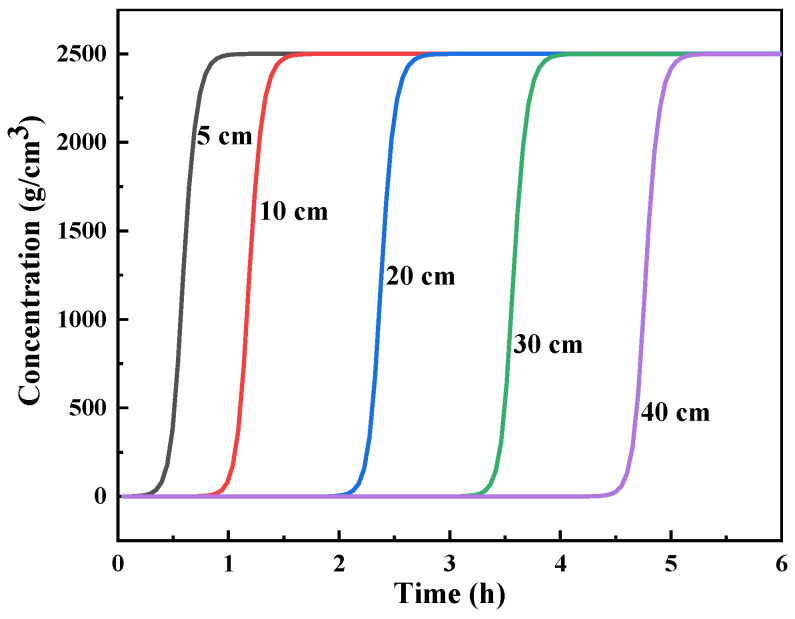
Predicted breakthrough curve of Sr(II) from the (DtBuCH18C6 + DBS + dodec)/SiO_2_-P column under various column lengths.

In the actual separation process, the increasing length of the adsorption column in simulation experiments would lead to problems such as a long reaction cycle time, lower experimental efficiency, and higher costs. This problem is especially serious in radioactive conditions, thereby limiting the development of a large number of experiments. The length of the column also affects the loading amount of the adsorbent and the column pressure. With the use of the constants from the above simulation, the breakthrough curves of different bed heights were calculated at a flow rate of 0.3 m^3^/min (Figure 6). Given the high affinity of crown ether for Ba(II), with the assumption that Ba was totally co-adsorbed as Sr in HLLW, the initial concentration of the feed solution was calculated as 2500 g/m^3^ (PWR, 235U:4.5%, burnup: 4.5 × 10^4^ MW/t, 5 years cooling down) [23]. As seen in Figure 5, the breakthrough curve was a regular shift with a column length change. The 5% breakthrough time increased from 0.43 to 4.6 h in column lengths ranging from 5 to 40 cm. The longer column contributed to a longer breakthrough time and a larger processing volume for one cycle. This result is similar to the results of Fujie et al. [41]—that is, a standard breakthrough curve would appear to move parallel to the time axial under constant flow velocity and sufficiently long column condition. Under specific experimental conditions, the appropriate design of the adsorption column length is of great significance for improving adsorbent utilization and economy. The column was proposed to have a length of around 40 cm, according to our previous study, so that the requirements of efficient processing and a relatively large processing volume could be optimized. The effect of the column’s height/diameter ratio and heat transfer should be clarified in detail in our subsequent studies.

#### 3.2.3. Dynamic Desorption Performance

**Figure 7:**
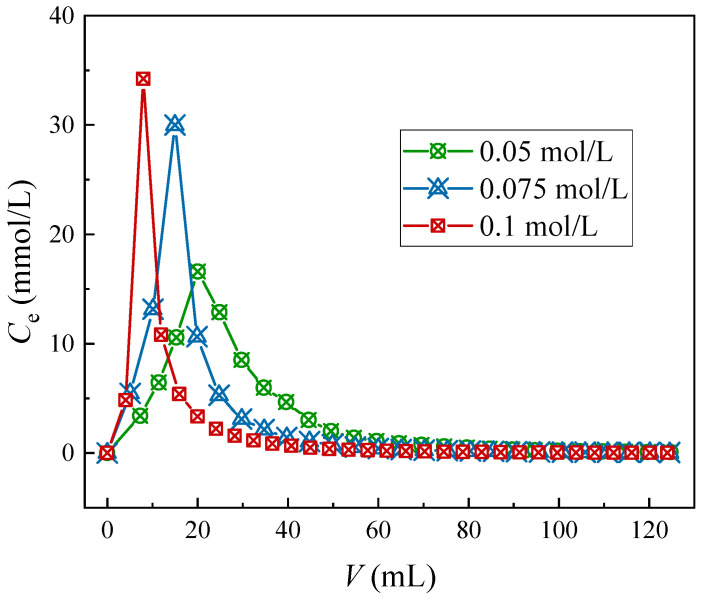
Sr(II) elution curves through different concentrations of Na-DTPA.

In accordance with the Sr(II) desorption performance of the considered four elutes to the (DtBuCH18C6 + DBS + dodec)/SiO_2_-P adsorbent in the above section, the Na-DTPA solution was utilized to further investigate the dynamic Sr(II) elution ability of the test column. The dynamic ionic elution properties of varying concentrations of the Na-DTPA solution to the adsorption column are described in Figure 7.

As the Na-DTPA concentration increases, the Sr(II) elution plots of the column tend to present with a sharper and stronger elution peak. At concentrations of 0.05, 0.075, and 0.1 mol/L, volumes of 94.7, 70.2, and 53.4 mL of the eluent were required to realize the complete elution of Sr(II). The elution rates achieved by the three Na-DTPA concentrations were 82.2%, 86.4%, and 91.2%, respectively. The experiment results indicated that increasing the concentration of Na-DTPA could be beneficial to enhance the efficiency of the elution process because of the improved complexion of Sr(II) with [DTPA]^5−^. Thus, the 0.1 mol/L Na-DTPA solution could be used in the design of the column separation process of HLLW to attain Sr(II) separation and recovery.

#### 3.2.4. Column Separation of Simulated HLLW

**Figure 8:**
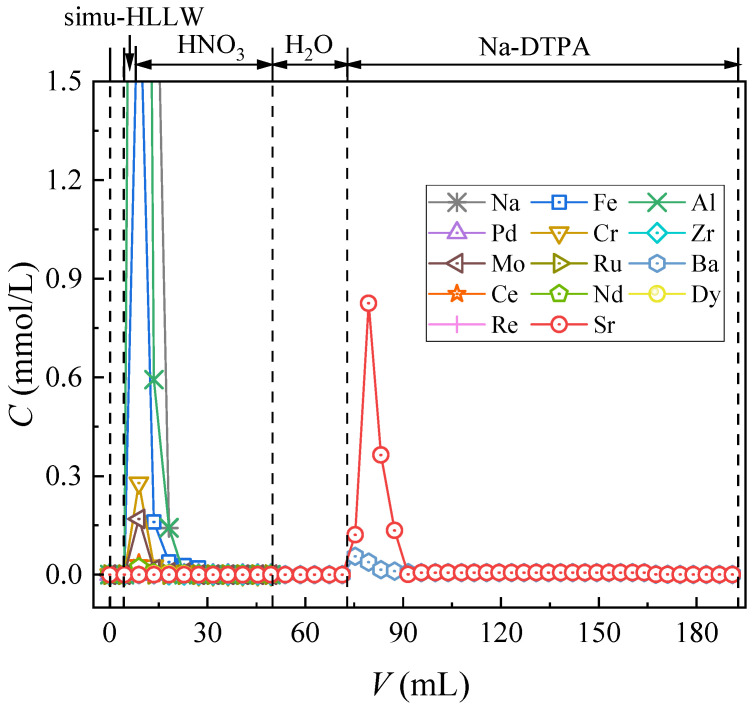
Chromatographic partitioning of Sr(II) from SHLLW by the (DtBuCH18C6 + DBS + dodec)/SiO_2_-P packed column.

Given the complex composition inside HLLW, the effect of competitive ions on the dynamic Sr(II) separation property of the material needs to be investigated. Therefore, SHLLW was used for the dynamic column separation study of the synthesized material, and the results are shown in Figure 8.

Aside from the elements of Sr(II) and Ba(II), all rested impurity nuclides were eluted from the adsorption column after 3 mol/L nitric acid solution feeding. The similar chemical properties of Ba (II) and Sr (II) led to the simultaneous adsorption of Ba (II) on (DtBuCH18C6 + DBS + dodec)/SiO_2_-P [36]. Flowing of a 20 mL nitric acid solution could successfully achieve the separation of Sr(II) with most coexisting cations in the SHLLW, indicating the excellent Sr(II) dynamic selective adsorption capacity of (DtBuCH18C6 + DBS + dodec)/SiO_2_-P. With the better elution efficiency of the Na-DTPA solution to Sr(II) under low acidity, the deionized water was retained for use after the feeding of HNO_3_ to decrease the H^+^ concentration of the filled layers. Sr(II) remained anchored in the column after the offering of water proved that the modification of DBS resulted in a wider acidity range of DtBuCH18C6-loaded SiO_2_-P material, and the adsorbent exhibited good Sr(II) adsorption performance at low acidity. The recovery rate of each element is shown in Table 5. All nuclides could achieve a recovery of more than 80%, and the rate of Sr(II) exceeded 99%, which revealed that Sr(II) could be successfully separated from HLLW through the (DtBuCH18C6 + DBS + Dodec)/SiO_2_-P adsorption column.

However, some Ba(II) impurities existed in the eluent during the Sr(II) elution process of the packed adsorption column by using the Na-DTPA solution, revealing that the synthesized adsorbent possessed a certain Ba(II) complexation capacity. This condition could be reasonably ascribed to the similar chemical properties of Ba(II) and Sr(II) and that the DtBuCH18C6 adsorbent has a poor ability to separate Ba(II) and Sr(II) elements.

### 3.3. Density Functional Theory Computational Results

**Figure 9:**
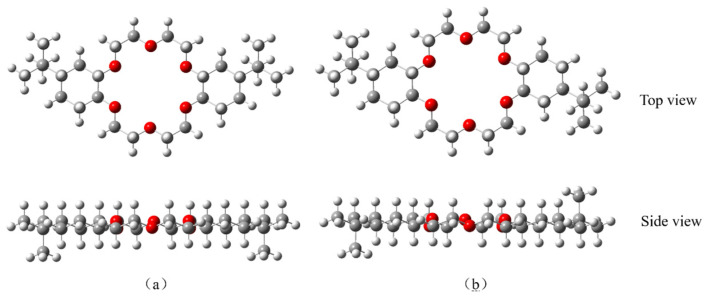
Built geometric models of 4,4′-di-tert-butylcyclohexane-18-crown-6 (**a**) and 4,5′-di-tert-butylcyclohexane-18-crown-6 (**b**).

**Figure 10:**
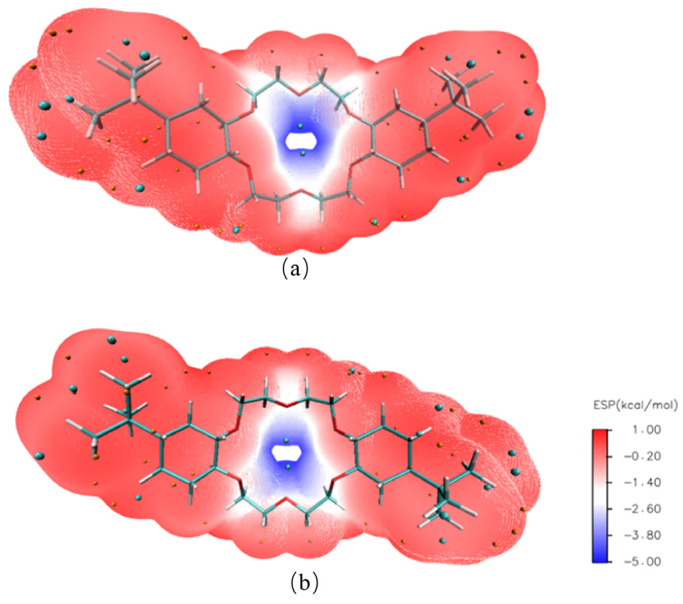
ESP results of 4,4′-di-tert-butylcyclohexane-18-crown-6 (**a**) and 4,5′-di-tert-butylcyclohexane-18-crown-6 (**b**).

Two geometric models of 4,4′-di-tert-butylcyclohexane-18-crown-6 and 4,5′-di-tert-butylcyclohexane-18-crown-6 structures were established by using GaussView 6.0 software to derive a further understanding of the complexation properties of DtBuCH18C6 to metal ions [42] (Figure 9). The ESP diagrams of the ground state obtained after the optimization of the built models are presented in Figure 10, and the red and blue regions in the picture indicate the positive and negative ESP areas of DtBuCH18C6, respectively. The centers of the DtBuCH18C6 structures exhibited a relatively low ESP, revealing the distribution of the main adsorption sites.

#### 3.3.1. DtBuCH18C6-Sr(II) Complexation Model

**Figure 11:**
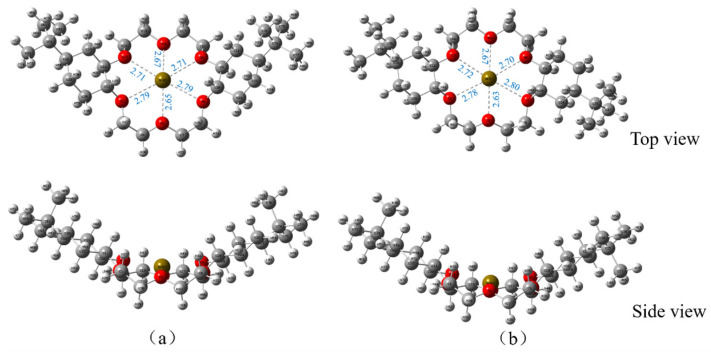
Optimized Sr(II) complexation models of 4,4′-di-tert-butylcyclohexane-18-crown-6 (**a**) and 4,5′-di-tert-butylcyclohexane-18-crown-6 (**b**).

On the basis of the ESP results of the optimized DtBuCH18C6 structure, DtBuCH18C6-Sr(II) complexation models were developed, and the Sr(II) atom was set in the center of the crown ether ring. Figure 11 displays the geometric optimized DtBuCH18C6-Sr(II) complexation models. The frequency calculation results showed that no virtual frequency existed, indicating the complexation models after optimization were the most stable structures. Sr(II) remained in the center of the DtBuCH18C6 ring and close to the oxygen atoms inside the crown ether ring, and the center-to-center distances of the Sr-O bonds varied from 2.63 Å to 2.80 Å, indicating that DtBuCH18C6 was mainly dependent on the adsorption sites of the oxygen atoms to realize the complexation ability of Sr(II). In addition, both optimized complexation structures were almost identical, presenting with unidirectional bending of the crown ether structure. This finding reveals the similar Sr(II) complexation capacity of the two DtBuCH18C6 structures.

#### 3.3.2. Mulliken Charge Distribution

The overall coordination numbers of Sr(II) inside both complexes were calculated by using the method introduced in Reference [43], and the results were 6-coordinate for the two structures. Localized orbital bonding analysis was utilized to determine the valence state of the atoms, and the Sr valence states obtained inside both complexes were +2 at a judgment threshold of 50%. These two calculation results further suggest interaction between Sr(II) and O atoms. Table 6 shows the atoms’ Mulliken charge distribution of DtBuCH18C6-Sr(II) complexes in the water and gas phases to quantify the charge change of both complex structures with atomic displacement. The Sr atom has a positive charge, and all O atoms have negative charges with similar values, thereby revealing the similar transfer charges number from the oxygen ligand of the crown ether ring to the center atom. Moreover, the interaction forces of Sr(II) with O atoms in different positions were close. The net charge of Sr(II) inside the water phase is greater than that in the gas phase, indicating a greater transfer charge number from the oxygen ligand to Sr(II) in the gas phase.

#### 3.3.3. Binding Energy Analysis

The quantified calculated energies of Sr(II) adsorption by DtBuCH18C6 are listed in Table 7. The results showed that the total energy of the system decreased after the formation of the DtBuCH18C6-Sr(II) complexes, indicating that the yield process of the complexes was energetically favorable [35]. Moreover, the binding energy is closely related to the stability and binding ability of the complex. The crown ether structure of the 4′,4″ isomer processed a lower adsorption energy compared to the 4′,5″ type, indicating that the stability of the complex that formed after the adsorption of Sr(II) by 4′,4″-Di-tert-butyldicyclohexyl-18-crown-6 was stronger. However, the interaction free energies were similar, further proving that the Sr(II) complexation ability of the two crown ether structures was similar.

#### 3.3.4. Anionic Coordination of DtBuCH18C6-Sr(II)

**Figure 12:**
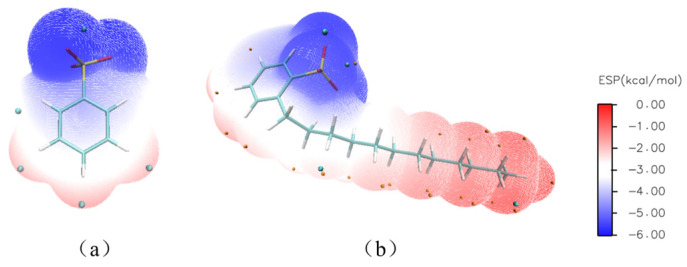
ESP graphics of benzenesulfonic acid (**a**) and DBS (**b**).

During the Sr(II) extraction process of DtBuCH18C6, the nitrate ions and DDBS inside the aqueous solution could be the ligand ions that participate in the complexation reaction. Therefore, two complexation models of DtBuCH18C6 to Sr(II) with the coordination of different ions were developed to investigate the effect of ligand ions on the complexation process. The ESP graphics of benzenesulfonic acid (BSA) and DBS were calculated (Figure 12), including the HOMO–LUMO gap and the atomic dipole moment corrected Hirshfeld (ADCH) atomic charges, to simplify the calculation process (Table 8). The results showed that the gap values, ADCH atomic charges, and ESP images of both ligands were relatively close, indicating that using BSA to replace DBS as the ligand for the complexation calculation is reasonable.

**Figure 13:**
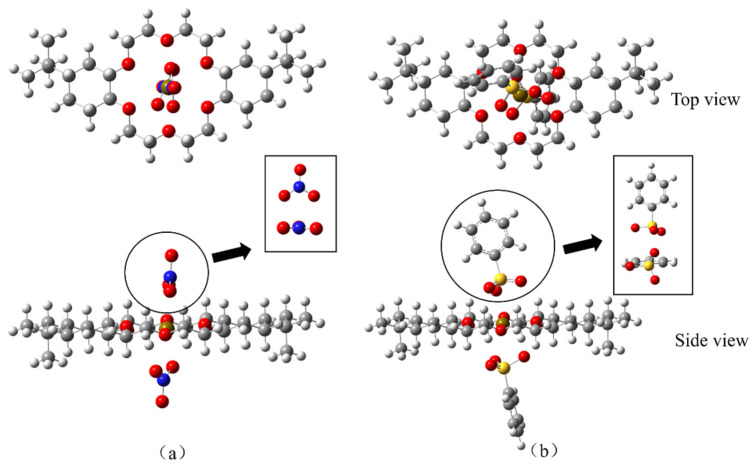
Geometric models of DtBuCH18C6-Sr(II) coordinated with the nitrate ligands (**a**) and BSA (**b**).

**Figure 14:**
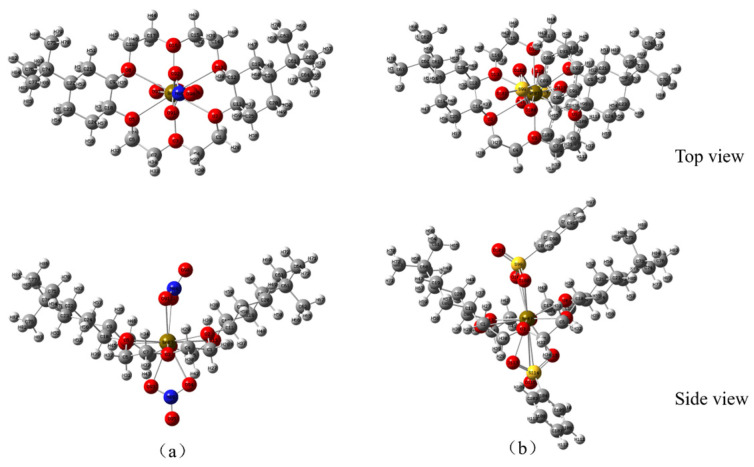
Optimized models of DtBuCH18C6-Sr(II) coordinated with the nitrate ligands (**a**) and BSA (**b**).

On the basis of the ESP mapping images, the ionic coordination models of DtBuCH18C6-Sr(II) with BSA or the nitrate ligands (Figure 13) were established, and the optimized structures of the two coordination models are shown in Figure 14. The frequency calculation results showed no virtual frequency, indicating that the optimized model possessed the most stable structure. Therefore, both DBS and nitrate ions can be complexed with DtBuCH18C6-Sr(II) through a bidentate coordination mode. With the exception of the misattributed C atoms, the total coordination number of Sr(II) inside each coordination model was calculated to be 14, including 10 O atoms and 2 S or N atoms, which is consistent with the optimized structure.

#### 3.3.5. Interaction Force and Binding Energy Analysis

**Figure 15:**
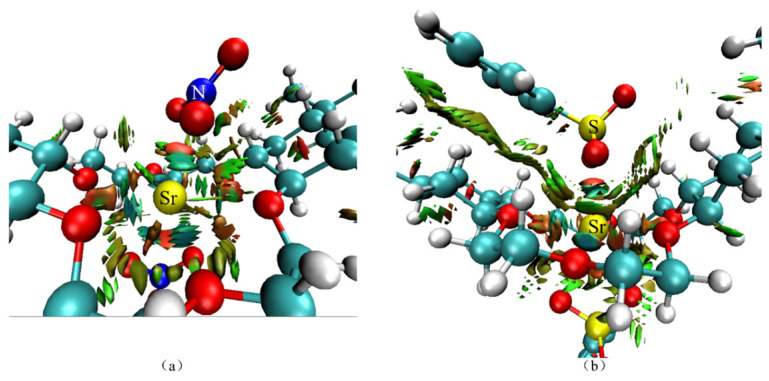
Theoretical calculation RDG results of DtBuCH18C6-Sr(II) coordinated with nitrate ligands (**a**) and DBS (**b**).

Figure 15 shows the reduced density gradient (RDG) isosurface of the center of the DtBuCH18C6-Sr(II) complexes with BSA or the nitrate ligand coordination. The results indicated that weak interaction attractions existed between Sr(II) and the O atoms of the ionic ligand, and a weak repulsion existed between Sr(II) with the N or S atoms. The direct van der Waals force interactions were suggested to occur between the benzene ring of DBS and the crown ether.

Thermodynamic calculations for both complexes were performed at the def2-svp basis group level, and the results are listed in Table 9. The negative reaction energies indicated that both coordination reactions were energetically favorable for the formation of stable complex structures. Therefore, the promotion mechanism of DBS could be DBS as a ligand that participated in the complexation process of DtBuCH18C6 on Sr(II), thereby enhancing the complexation adsorption ability of the crown ether groups on Sr(II) in the aqueous solution. Furthermore, DBS, as an organic BSA, exhibited certain hydrophobicity [44,45], and the complexation behavior of DBS with Sr(II) could explain the weak desorption ability of deionized water and nitric acid to (DtBuCH18C6 + DBS + Dodec)/SiO_2_-P adsorbent after Sr(II) adsorption. The interaction free energy of the crown ether coordinated with nitrate showed a greater value than that with BSA, revealing that the interaction of the DtBuCH18C6-Sr(II) complex with the nitrate ion was stronger than that of DBS.

## 4. Conclusions

Static and dynamic selective removal performance studies of a DBS-modified silica-based DtBuCH18C6 adsorbent for Sr(II) in simulated HLLW revealed that the synthesized material had excellent Sr(II) separation ability and could be used for Sr(II) decontamination in HLLW. (DtBuCH18C6 + DBS + dodec)/SiO_2_-P achieved 88.2% adsorption column utilization during the dynamic test at a flow rate of 0.3 cm^3^/min. A mathematical adsorption dynamics model was built: a standard breakthrough curve would appear to move parallel to the time axial under a constant flow velocity and sufficiently long column condition. The close values of the 5% and 100% breakthrough periods from model fitting and the experiment data proved the feasibility of a reasonable mechanical model and appropriate calculations of the parameters. Chromatographic separation of Sr(II) from the SHLLW was accomplished by using stepwise elution techniques. The Sr(II) ions were effectively eluted with 0.1 mol/L Na-DTPA, and the dynamic recovery was up to 99%. The optimized structures and interaction energies of DtBuCH18C6 coordinated with Sr(II) and the DBS modifier were investigated by using DFT calculations. The adsorption sites of oxygen atoms played a major role in the complexation process, and the center distance of the Sr-O bond in the optimized complexation model was 2.63 Å to 2.80 Å. The crown ether could complex with Sr(II) to form a stable structure under the coordination of DBS, indicating that DBS has a facilitating effect on the Sr(II) complexation process.

## Figures and Tables

**Table 1:** Composition of SHLLW.

Element	Conc. (g/L)	Element	Conc. (g/L)
Na	31.2 g/L	Pd	0.05 g/L
Nd	0.7 g/L	Re	0.2 g/L
Ce	0.8 g/L	Ru	1.2 g/L
Cr	1.1 g/L	Sr	0.9 g/L
Dy	0.1 g/L	Zr	0.1 g/L
Mo	1.2 g/L	Al	5.3 g/L
Fe	4.8 g/L	Ba	1.1 g/L

**Table 2:** Dynamic binding capacity and the column utilization.

Flow Rate (mL/min)	5% B. P. (BV)	5% B. Cap. (mmol/g)	100% B. P. (BV)	T. Cap. (mmol/g)	Utilization (%)
0.3	13	0.22	25	0.24	88.17

**Table 3:** Calculated mass coefficient *K*_fa_ and parameters of the Freundlich model.

DtBuCH18C6 + DBS + Dodec			Silica Support		
*K_fa_*_1_	*K_F_*_1_	*b*_1_	*K_fa_*_2_	*K_F_*_2_	*b*_2_
142.5	3.1011	0.3818	10	0.055	0.2

**Table 4:** Comparison of the predicted breakthrough curve and actual experimental result.

Breakthrough Points	5% BP		100% BP	
Flow rate (mL/min)	0.3	0.5 [23]	0.3	0.5 [23]
Simulation result (h)	2.72	2.03	6.83	5.20
Experiment data (h)	3.05	2.56	6.92	4.53

**Table 5:** Recovery rate of each ion inside the SHLLW.

Elements	Recovery (%)	Elements	Recovery (%)	Elements	Recovery (%)
Sr	>99	Pd	>99	Ce	>99
Ba	>99	Cr	>99	Nd	>99
Na	82.35	Zr	98.88	Dy	>99
Fe	>99	Mo	>99	Re	>99
Al	>99	Ru	96.42		

**Table 6:** Mulliken charge distribution of the DtBuCH18C6-Sr(II) complex.

Atoms	4′,4″		4′,5″	
	Gas	Water	Gas	Water
Sr	1.474	1.670	1.474	1.669
O2	−0.480	−0.427	−0.449	−0.429
O3	−0.466	−0.433	−0.460	−0.438
O5	−0.480	−0.427	−0.455	−0.427
O8	−0.454	−0.453	−0.480	−0.453
O13	−0.455	−0.453	−0.484	−0.452
O16	−0.460	−0.434	−0.466	−0.432

**Table 7:** Quantified calculated energies for the DtBuCH18C6-Sr(II) complexes.

Energy	4′,4″	4′,5″
*E_complex_* (Hartree)	−1577.5760	−1577.5758
*E_DtBuCH_*_18*C*6_ (Hartree)	−1546.9517	−1546.9517
*E_Sr(II)_* (Hartree)	−30.5711	−30.5711
*E_BSSE_* (Hartree)	0.0014	0.0014
Δ*E* (kJ·mol^−1^)	−135.9013	−135.5275

**Table 8:** HOMO–LUMO gap and ADCH atomic charges of the DBS and BSA ligands.

	DBS	BSA
HOMO–LUMO gap/eV	7.866	7.665
ADCH atomic charges/Angstrom		
S	0.666	0.661
O1	−0.463	−0.512
O2	−0.446	−0.512
O3	−0.508	−0.494

**Table 9:** Calculated energies for DtBuCH18C6-Sr(II) coordinated with DBS or the nitrate ligands.

Energy	NO_3_^−^	C_6_H_5_O_3_S^−^
*E_complex_* (Hartree)	−2137.8550	−3287.3322
*E_DtBuCH_*_18*C*6_ (Hartree)	−1546.9517	−1546.9517
*E_Sr(II)_* (Hartree)	−30.5687	−30.5687
*E_anion_* (Hartree)	−279.4531	−854.7269
Δ*E* (kJ·mol^−1^)	−861.5328	−189.7551

## Data Availability

Data are available from the corresponding author by request.
